# 5. Prehospital management

**DOI:** 10.1007/s00068-025-02825-7

**Published:** 2025-05-06

**Authors:** Andrej Čretnik, Roman Pfeifer

**Affiliations:** 1 Department of Traumatology, University Clinical Center Maribor, Maribor, Slovenia; 2 Department for Traumatology, University Hospital Zurich, Zurich, Switzerland

**Keywords:** ESTES, Polytrauma, WhiteBook

## Abstract

This chapter outlines the essential requirements for emergency responses to severe injuries. It emphasises the critical steps healthcare professionals must take in urgent situations, including: Rapid assessment and triage to prioritise treatment; Techniques for controlling massive external haemorrhages to prevent life-threatening blood loss; Maintaining a clear airway, ventilation, and neck stabilisation to support breathing and minimise spinal injury risks; Intravenous fluid replacement and medication administration to stabilise patients' conditions; Proper immobilisation of injuries to prevent further harm during transportation; Facilitating rapid and effective transfers to specialised medical centres, with clear communication ensuring seamless continuity of care. By adhering to these protocols, healthcare providers can efficiently navigate emergency situations, saving lives and minimising the long-term impact of critical injuries and illnesses.

## Organisation

The management of severely injured patients has evolved significantly over recent decades due to improvements in pre-hospital organisation, on-site assessment, initial care, and transportation protocols. The process begins at the trauma site and requires an interdisciplinary approach.

The initial “Chain of Rescue” consists of:

- Pre-hospital care
- Accident and Emergency Unit
- Damage control (emergency) interventions

Beside the type of injury (e.g., head injuries and severe bleeding), pre-hospital time is the most critical independent factor predicting mortality. The treatment approach also depends on transportation time. In urban settings (short transportation time), a “scoop and run” or “load and go” concept is preferred. In rural settings (long transportation time), a “stay and play” concept may be necessary.

### Note

The balance between “load and go” and “stay and play” approaches must prioritise the urgency of life-saving interventions. While critical interventions such as airway management or haemorrhage control may require immediate action at the scene and should not be deferred to reduce pre-hospital time, certain measures like surgical haemostasis of internal bleeding are best performed in hospital settings.

## Transfer of information and triage

### Alarming and notification

- 112 is the universally accepted emergency number within the EU (European Union) (calls to 911 should be automatically redirected to 112).
- Communication systems must include a phone line (cable/optical fibre), wireless (GSM), and radio line, with at least one alternative system available.
- The dispatch centre (local or regional) is the primary point of coordination for alarming and resource management.

### Arrival and scene information

- Effective organisation and command must be established (e.g., a team commander, clearly marked, with mandatory radio communication).
- Scene security and safe access must be coordinated with relevant authorities:
  o Fire services for traffic accidents, building collapses, poisoning, and entrapped patients.
  o Police for demonstrations, dangerous situations, tear gas, or smoke.
  o Special Forces: For armed conflicts (e.g., AMOK incidents) or terrorist attacks.
- Access to victims is permitted only after a “safe approach” has been declared.
- The triage system should be adapted to the number of victims, available rescue teams, and situational protocols (e.g., major incidents, hazardous material (HAZMAT) events).

### Actors at the scene

- First responders: Act based on instructions from the dispatch centre or the scene commander after notification.
- Rescue teams: May include paramedics, emergency medics, and other pre-hospital personnel.
  o Teams without a physician can provide Basic Life Support (BLS) or partial Advanced Life Support (ALS).
  o Teams with an emergency physician can apply principles of Pre-hospital Trauma Life Support (PHTLS) or Advanced Trauma Life Support (ATLS).
- Command Coordination: When multiple teams are on-site, the most experienced person or physician should oversee coordination and command.

### Triage in mass casualty events

- When sufficient rescue resources are available, treatment should follow the principle “treat first what kills first” using PHTLS/ATLS (ABCDE) protocols?
- In mass casualty scenarios, TRIAGE systems and zones must be established for patients based on severity:
  o Green: Minor injuries.
  o Yellow: Moderate but non-life-threatening injuries.
  o Red: Life-threatening injuries requiring immediate intervention.
  o Black: Deceased or non-survivable injuries.
- Triage should incorporate primary (SIEVE ((Secondary Triage)) triage and secondary (SORT ((Secondary Organ Rescue Triage)) triage, based on physiological parameters (e.g., respiratory rate, systolic blood pressure, Glasgow Coma Scale) and anatomical considerations.

## Initial assessment and management requirements

A time-critical approach saves lives. The goals are hospital admission within 60 min and definitive clinical treatment within 90 min. Achieving these goals may be challenged by issues such as entrapped patients, prolonged rescue or transportation times, and other complicating factors.

Life saving measures must always take ultimate priority. If impaired vital functions are present in polytrauma patients, interventions should be performed as soon as possible, following PHTLS/ATLS principles—either at the scene after rescue or during transport:

Additional measures include:

- Positioning and immobilisation of patients (e.g., using spinal blocks, spine boards, stretchers, or splints).
- Early initiation of care for thermal injuries and burns.
- Identification and management of concomitant injuries (e.g., deafness as a potential symptom of a blast syndrome).
- Collecting information on pre-medications (e.g., anticoagulants) whenever possible.

The ultimate goal to reduce mortality is timely transport to an appropriate trauma hospital. Criteria for transportation to different categories of hospitals (as detailed in Chapter 4) should align with internationally accepted standards.

## Transportation modalities

- Ground-based transport: Severely injured patients can be transported by regular ambulances staffed with medically trained personal (paramedics) or physician-equipped ambulances.
- Air transport: Helicopters are effective for primary transport, particularly in rural areas with long transportation times, and for secondary interhospital transfers. However, their use may be limited by darkness and adverse weather conditions.
- Central Coordination of Transport: Transportation should be centrally directed (e.g., via dispatch with active communication). This is especially critical during major or mass casualty incidents to prevent overloading certain hospitals or inappropriate transfers to facilities lacking the necessary diagnostic and therapeutic capabilities (e.g., neurosurgery, cardiovascular or thoracic surgery).

When there is sufficient capacity, transport should be prioritised as follows:

- Red → Yellow → Green patients.
- In officially declared “decompensated” situations”, priority should be given to patients with the greatest chance of survival.

A unified telematics system for data exchange and cross-border assistance could help avoid transport inefficiencies and ensure equitable access to care.

## Conclusion and needs for the future

To ensure optimal outcomes for severely injured patients, more uniform organisation and appropriately equipped and trained rescue teams are needed in the pre-hospital setting, which serves as the first link of the rescue chain. Continuous implementation and evolution of PHTLS and ATLS principles are essential. Further development and modernisation of equipment for pre-hospital care is required. A unified telematics system for data exchange and medical coordination within and across EU countries should be established to improve outcomes and ensure seamless cooperation.

## Data Availability

No datasets were generated or analysed during the current study.
